# A Pilot Study on the Feasibility of Developing and Implementing a Mobile App for the Acquisition of Clinical Knowledge and Competencies by Medical Students Transitioning from Preclinical to Clinical Years

**DOI:** 10.3390/ijerph19052777

**Published:** 2022-02-27

**Authors:** Alvaro Prados-Carmona, Francisco Fuentes-Jimenez, Rafael Roman de los Reyes, Antonio García-Rios, Jesus Rioja-Bravo, Ezequiel Herruzo-Gomez, Pablo Perez-Martinez, Jose Lopez-Miranda, Javier Delgado-Lista

**Affiliations:** 1 Department of Dermatology, Hospital Universitario San Cecilio, 18016 Granada, Spain; apradoscar@gmail.com; 2 Maimonides Institute for Biomedical Research Córdoba, 14004 Cordoba, Spain; fjfuentesjimenez@yahoo.es (F.F.-J.); angarios2004@yahoo.es (A.G.-R.); jlopezmir@uco.es (J.L.-M.); 3 Department of Medical and Surgical Sciences and Grupo Docente 123, Universidad de Cordoba, 14071 Cordoba, Spain; 4 Lipid and Atherosclerosis Unit, Department of Internal Medicine, Hospital Universitario Reina Sofía, 14004 Cordoba, Spain; 5 CIBER Fisiopatologia Obesidad y Nutricion (CIBEROBN), Instituto de Salud Carlos III, 28029 Madrid, Spain; 6 Advanced Computer Architecture Group, Department of Computer Architecture, Electronics and Electronic Technology, Universidad de Cordoba, 14071 Cordoba, Spain; i62rolor@uco.es (R.R.d.l.R.); i52ribrj@uco.es (J.R.-B.); 7 Department of Computer Architecture, Electronics and Electronic Technology and Grupo Docente 30, Universidad de Cordoba, 14071 Cordoba, Spain; el1hegoe@uco.es

**Keywords:** medical education, educational models, smartphone, cardiology, clinical competence, satisfaction

## Abstract

Due to the COVID-19 pandemic and the consequent restrictions, universities have had to adapt their curricula substantially to new schemes in which remote learning is of the essence. In this study, we assess the feasibility of developing a mobile app supplementary to the distant teaching paradigm for the “Cardiology” module of the “General Pathology” subject in undergraduate Medical Education, and we evaluate its impact and acceptability. A cohort of volunteer second-year medical students (*n* = 44) had access to the app, and their opinions on its utility (1–10) were collected. Additionally, the students were invited to refer their expected satisfaction (1–10) with a blended learning methodology overlapping this new tool with the traditional resources. The average expected satisfaction had been compared to the average satisfaction obtained by just the traditional methodology in other modules from the same subject. Through a qualitative approach, we defined the strengths and weaknesses of the tool. Seventy-seven percent of the participants rated at 8/10 or more the potential learning value of the application and, if used as a supplement to traditional teaching, it would also statistically improve the satisfaction of students (6.52 vs. 8.70, *p* < 0.001). Similarly, the qualitative data corroborated the benefits of such innovation. Multidisciplinary collaborations are encouraged to develop teaching innovations, although further research should aim to better define the effectiveness of learning with these resources.

## 1. Introduction

Since the early days of modern Medical Education and the publication of the Flexner Report [1], our training has been focused not only on “learning” but on “learning how” [2]. Nowadays, even more importance is given to the acquisition of competencies, and terms such as Competency-Based Medical Education (CBME) are deeply established [3]. However, there are huge challenges that make it difficult to effectively put these models into practice and involve students in the clinical environment [4], such as logistics, work overload of the clinical staff [5], the central role of the patient in the healthcare setting, etc. Additionally, the situation has dramatically shifted to an even more complicated scenario due to the COVID-19, with far-reaching consequences in education, over and above its economic repercussions and the impact on mental health [6,7]. Because of the COVID-19 pandemic, medical students in Spain were not allowed into health care institutions during the rest of the academic year 2020, and teaching had to be adapted to ensure remote learning. The concern about the disruption to the medical education process has been echoed all over the globe as social distancing became mandatory to curb the different waves of the pandemic. Now, despite returning to in-person teaching following careful consideration of local factors, some restrictions are still in place in hospitals, and the negative impact of the pandemic is expected to persist for a long time [8]. Patient care remains the top priority, and there is an attempt to minimize nonessential staffing in clinical environments [2,9]. In this “new normal” situation with an uncertain future and increased patient demands, we can predict that clinical rotations will still be sporadically deferred as quarantines and social distancing measures are occasionally required [10]. Therefore, students could find it difficult to accumulate enough hours at the bedside of the patients for which there is no apparent substitute [11]. While it is easier for preclinical students to transition from lectures to online platforms, remote learning is more challenging for students acquiring clinical competencies—including nontechnical, behavior, and communication skills—in their basic clinical placements or their core clerkships [12,13]. What is more, patient exposure in the different wards is essential for fostering students’ interest in the various specialties [14], and some countries cannot afford delays in incorporating the next generation of doctors into the hospitals [15].

In terms of cardiovascular semiology and its exploration, the main sources of information used until now have been traditional resources such as reference books that define the system of the exploration and the possible findings [16]. Competencies are supposed to be acquired through clinical placements (approximately 15% of the hours of the subject). This model is quite limited and progressively becoming outdated, and that is why many students also use third parties’ materials to complement their clinical hours [17]. This landscape has become even worse after the COVID-19 pandemic and the recurrent stay-at-home orders aimed at mitigating the spread of the virus. Students have been left seeking unorthodox opportunities to replace what they are missing from their clinical duties [14,18]. Unfortunately, this is performed without any guidance or feedback from faculty, lacking any possibility to track and supervise their autonomous learning [19]. However, in a context such as the one we are living in now, adapting to the need of the hour is imperative. There have been many examples in history whereby dealing with unprecedented challenges has led to improvements in how we understand education or healthcare [10]. This might also be one of those seminal moments in which we have to step up and find new ways of helping students acquire competencies from a distance, promote their engagement, and allow interaction between them and the university staff [13]. To accomplish this, we should aim to design and implement alternative learning streams beyond the traditional paradigm ensuring the integrity and continuity of the medical education process.

The positive impact of new technologies has been widely described [20], including simulators [21], for the acquisition of knowledge [22], competencies, and for building up confidence [23] in trainees in a cost-effective manner for the education [24] and public health systems [25]. Also, the preferences of new generations for these technologies have been exhaustively characterized [26,27,28,29]. There may be countless ways to implement already available technologies in learning. One of the recently proposed models has been ‘Mobile Learning’ (M-Learning), understood as the methodology that incorporates portable electronic devices to the teaching process inside and outside the classroom, focusing on the mobility of the learner—not in vain, attendance to lectures was declining even before the pandemic [6,13,29]. A blended learning methodology would be the one in which these new technologies are integrated with the traditional instructor-led and in-person activities. While it has been gaining increasing interest in the last years, SARS-CoV-2 has suddenly boosted the need to enrich this approach to education [28], mainly because the pandemic and the subsequent restrictions are unlikely to completely disappear in the foreseeable future [30]. In any case, new pathways to learning may be useful when normality is restored if they have not been envisioned only as crisis–response methods. This being so, these challenging times for students and all the different stakeholders involved in education might be sowing the seeds for sustainable innovations and new opportunities [31]. 

The implementation of new teaching schemes implies a drastic change and the investment of multiple resources [32]. Previous reviews did not provide substantial evidence [33,34], and qualitative analyses supporting new methodologies are also scarce [35]. However, it has been suggested that, for students, these models might prove advantageous in enhancing autonomous learning [36,37]. Prompted by the COVID-19 situation, our group has put in practice a pilot experiment to understand if these new resources could be useful in mitigating the consequences arising from the lack of teaching in the clinical setting [38] and whether students might want to continue using them in the future. 

Our group has created a mobile app to supplement remote teaching in the cardiovascular module for “General Pathology”, a subject of the second year of the Medicine Degree in the University of Cordoba aimed to introduce students to the physiopathology of the different body systems. The app was intended to provide the students with a virtual environment for training without time and place restrictions [8] and also without the consequences of negative evaluation [39]. Additionally, the app would allow faculty members to follow student’s progress over time and provide feedback if needed [21]. All its content was either created or carefully selected among high-quality, publicly available, and open access sources, as it has been encouraged during this pandemic not only for undergraduate Medical Education but also for residency programs and specialties such as Otolaryngology [14], Dermatology [40] and others. 

The first aim of this work was to create the app, evaluate the learning potential attributed to it by the students and, although beyond the scope of this paper, track their autonomous distance learning process. Secondly, we have assessed whether students’ satisfaction would increase in the future if the app were kept as a supplement to traditional teaching following an ‘M-Learning’ or blended scheme as defined above.

Our work stands out for being a pioneer in our environment and for managing to give a quick answer to the needs of health education institutions through a novel app created ad hoc for the purposes of the module [41] that can be downloaded and installed on the student’s smartphone.

## 2. Materials and Methods

A quasi-experimental, nonblinded, prospective intervention was carried out in a pool of second-year medical students at the University of Cordoba after the restriction on teaching in clinical settings were implemented due to the COVID-19 pandemic since March 2020. 

The study population is Medical students transitioning from foundation to clinical years, lacking enough access to the healthcare environment for appropriate acquisition of competencies. 

Inclusion criteria were:

– Enrollment in the subject “General Pathology” from the second year of Medicine Degree in our University;
– Voluntary provision of explicit consent;
– Owning an Android^®^ device connected to the internet.

The exclusion criteria were the impossibility to download the app throughout the totality of the follow-up period or the impossibility to use it.

The app, used to support remote learning during the cardiovascular module of “General Pathology”, included the following sections:

(i) Lectures and slides covering different topics in PDF format;
(ii) Physical exploration guide with an interactive auscultation module;
(iii) Main investigations in cardiovascular pathology with resources in various formats;
(iv) Tests for self-assessment;
(v) Arena: programmed team-based competition encouraging students to answer multiple-choice questions, thus reinforcing learning through the teaching period by engaging among peers.
(vi) Others: quick access to the university platform, results from tests, technical support, etc.

More information about the app code and content can be found in Appendix A. A link to an explanatory video can be found as Supplementary Material.

The design of the app was carried out by a workgroup from the University of Cordoba. It was created using Flutter (Google LLC., Mountain View, CA, USA); coded in Dart; graphically mocked-up with Figma (Figma Inc., San Francisco, CA, USA); distributed through Play Store (Google LLC., Mountain View, CA, USA). A “how to use” tutorial was uploaded to YouTube (Google LLC., Mountain View, CA, USA), and some notifications were shared with the student through Twitter (Twitter Inc., San Francisco, CA, USA). The storage of the data generated was based online (Firebase, San Francisco, CA, USA). Requirements of the app: Android^®^ operating system 5.0 or above. User registration process required institutional login and a personal code provided individually only to those students who volunteered to participate in the study.

After finishing the teaching period for the cardiovascular module, all participants were invited to share their opinions on this resource (*n* = 44), including both a quantitative and a qualitative approach to the utility and potential benefits of the new tool, to determine its feasibility and suitability. Opinions were self-collected, through specifically created online forms, without the intervention of the authors. The questions were designed to cover the aspects previously identified of higher interest through a review of the current literature regarding curricular adaptations after the COVID-19 pandemic and M-Learning methodologies. The survey was composed of a series of Likert-style questions as usual in the field. 

More information about the survey can be found in Appendix B. 

Additionally, participants were asked about their hypothetical satisfaction degree if the app was kept as a complement to traditional teaching. This was done using a one-to-ten scale. The average of the expected satisfaction obtained was later compared with the average satisfaction reported by the same students for previous modules of the subject taught without any supplement to traditional resources. Accepting an alpha risk of 0.05 and a beta risk of 0.2 in a two-sided test, 21 individuals were necessary to recognize as statistically significant a difference greater than or equal to 2 units. The standard deviation was assumed to be up to 3, and the anticipated dropout rate was fixed at 15%.

Secondary descriptive variables have also been collected from participants (sex, self-reported digital competency, the sufficiency of the information received about the tool, results obtained, etc.).

Data were processed and analyzed with SPSS v.24 (SPSS, Chicago, IL, USA). The sample has been described according to the distribution of secondary variables. Central tendency measures have been accompanied by their corresponding dispersion measure: Mean (±Standard Deviation). Sometimes minimum and maximum values were indicated when they proved relevant. There were no missing values. All the confidence intervals were estimated at 95%. All the contrasts were bilateral (two-sided), and those with *p* < 0.05 were considered significant. Test statistics values were reported in case of significant results. Cohen’s d was used for reporting the effect size where relevant. For bivariant analysis, proper parametric or nonparametric tests were used depending on whether the data distributions were normal or not (according to the Shapiro–Wilk test’s result when *n* < 30).

(a) For comparing quantitative variables, we used:
  – Contrasts between two groups with independent/unpaired data: Student *t*-test (parametric) or Mann–Whitney *U*-test (nonparametric);
  – Contrasts between two groups with paired data: Student *t*-test for paired data (parametric) or Wilcoxon test (nonparametric).
(b) For correlating quantitative variables, Pearson’s linear correlation coefficient (r) was used.

Ethical aspects:

This study was conducted following the Declaration of Helsinki and according to national and international policies. Explicit informed consent was needed to validate the registration process of the participants for the use of the app.

## 3. Results

### 3.1. Participants’ Demographics and Previous Digital Competency

After presenting the project, 69 students volunteered and completed the registration process in the app. Of those, 50 were women (72.5%) and 19 were men (27.5%), resulting in a sex ratio (women/men) of 2.63. Forty-four (*n*) of the registered users answered the final survey. Table 1.

**Figure 1:**
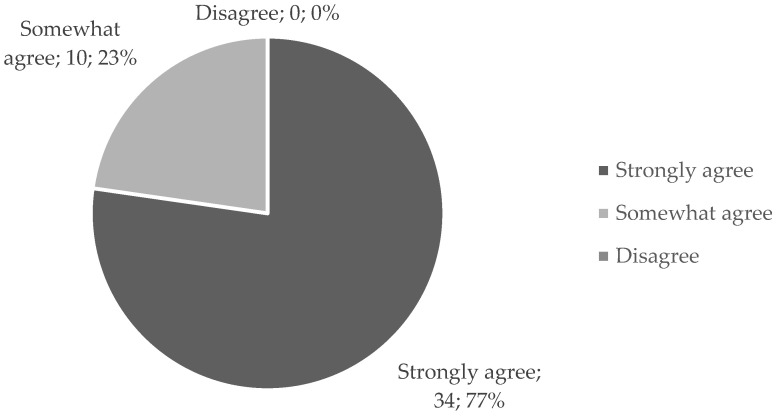
Students’ self-reported degree of digital competence indicated by declaring their level of agreement with the statement “My digital competence is good and I can use new technologies with ease” (level of agreement; the number of students; percentage of students).

Students were asked to self-report their previous digital competency by indicating to what extent they agreed with the following sentence: “My digital competence is good and I can use new technologies with ease”. Thirty-four students “strongly agreed”, ten “somewhat agreed”, and none of them “disagreed” with the statement. These results are shown in Figure 1.

Regarding the information provided to the participants about the app, its functionalities, and the purposes of the project, all participants (*n* = 44) considered it sufficient.

### 3.2. Team-Based Competition and App-Use Data

The mean student participation index was 32.37% (SD: 15.98) (the maximum participation rate was 50.76% and the minimum 21.73%). During the eight days of the competition, 1889 multiple choice questions were answered. 

### 3.3. Learning Value Attributed to the App

The participants were asked for their opinion on the potential learning value of the app regarding the content taught within that particular subject. 

**Figure 2:**
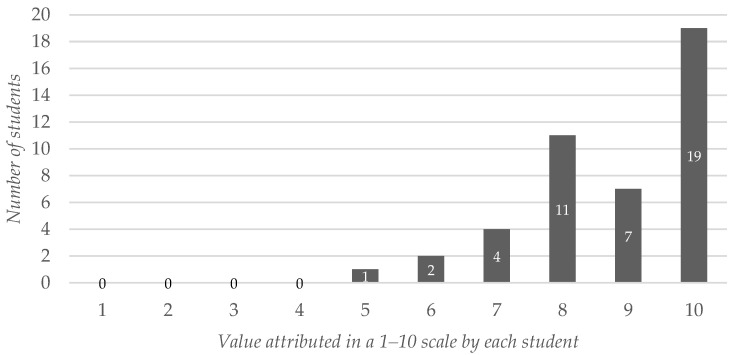
Students’ assessment of the app as a self-learning tool to improve students’ auscultation skills and competency in identifying heart sounds. The values are expressed on a 1–10 scale.

When we asked the students to what extent the app could improve the auscultations skills and the identification of heart sounds—promoted by enabling an interactive virtual patient—37 out of the 44 participants (84.09%) assigned a value of 8 or higher on the 1–10 scale. The average answer was 8.77 (SD: 1.34), shown in Figure 2.

**Figure 3:**
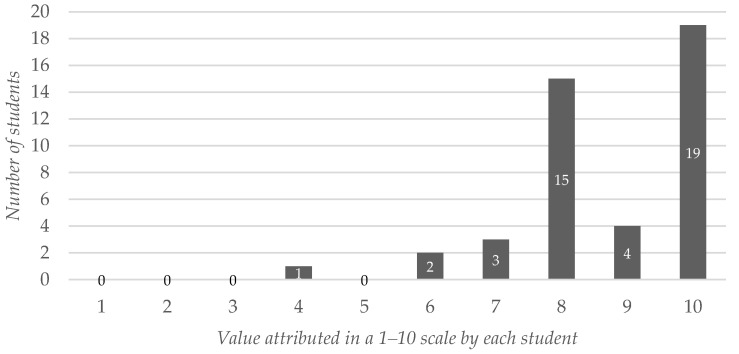
Students’ assessment of the app as a self-learning tool to improve students’ knowledge on the matter and other competencies covered in the subject. The values are expressed on a 1–10 scale.

Similarly, when answering the same question but referring to “general knowledge and competencies of the subject”, 38 out of the 44 participants (86.36%) answered with a value of 8 or higher. The average value attributed to the app as a tool useful for meeting the general requirements of the subject was 8.70 (SD: 1.40), shown in Figure 3.

### 3.4. Influence of Self-Reported Digital Competence on the Learning Value Attributed to the App

We placed 34 students who “strongly agreed” that their digital competency was good into the “high digital competency” or “A” group, and the 10 remaining students who “somewhat agreed”, into the “low digital competency” or “B” group. The mean of the potential learning value attributed to the app, both for the “auscultation skills” and for the “general knowledge and competencies of the subject”, were compared between the two groups. We did not find statistically significant differences between groups with different degrees of digital competence neither for “auscultation skills” (mean “A” = 8.76; mean “B” = 8.80; *p* = 0.943) nor for the “general knowledge and competencies” of the subject (mean “A”= 8.74; mean “B” = 8.60; *p* = 0.793).

### 3.5. Acceptability of the App

**Figure 4:**
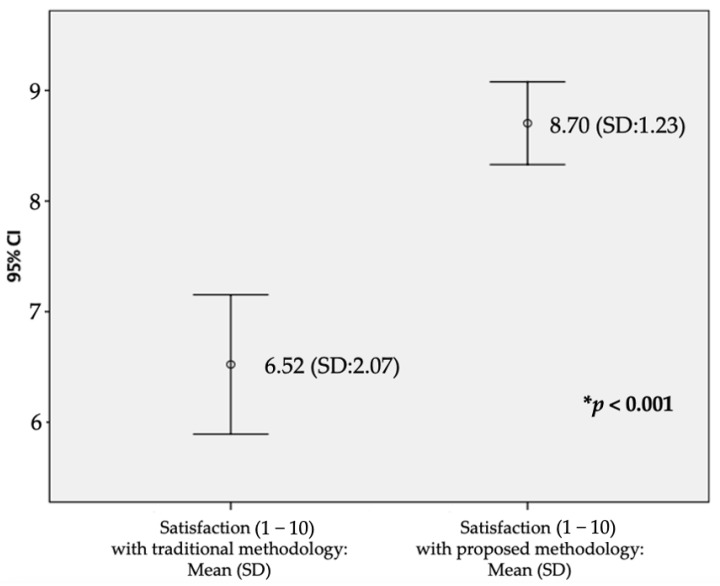
Satisfaction (1 − 10) of students with both teaching methodologies. Mean (SD). * The difference was found statistically significant.

To assess the acceptability of the app, the participants were asked about their expected satisfaction with a teaching methodology that included the app as a supplementary tool to lectures and hospital placements. Results were compared with the average satisfaction with the traditional teaching methodology applied to previous modules of the subject. The mean satisfaction of the students with the traditional methodology was 6.52 (SD: 2.07), and the mean expected satisfaction with maintaining the app as a complementary teaching method increased to 8.70 (SD: 1.23). This difference was found statistically significant (*p* < 0.001; t = −7.585; Cohen’s d = 1.28), Figure 4.

The different degrees of digital competence, as stated above, did not differ when comparing the expected satisfaction with the new methodology between the two groups of digital competence (mean “A” = 8.79; mean “B” = 8.4; *p* = 0.459).

### 3.6. Correlation between Satisfaction and Learning Value Attributed to the app

**Figure 5:**
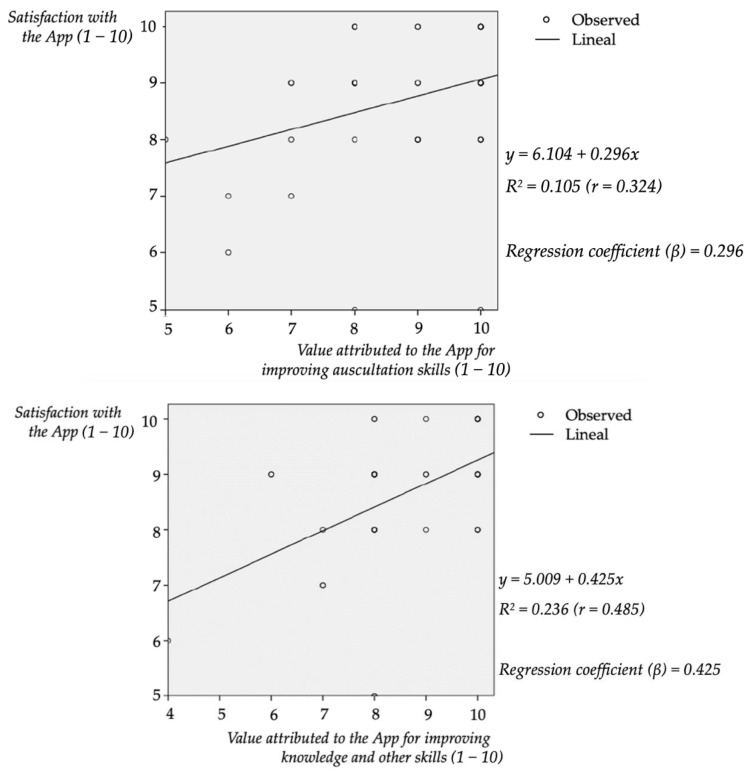
Regression lines correlating the expected satisfaction with a methodology that incorporates the app and the value attributed to it as a learning tool, first for “auscultation skills” and second for “general knowledge and other skills”.

We found statistical differences in the learning value attributed to the app (regarding “auscultation skills” and “general knowledge and competencies”) depending on the degree of expected satisfaction reported by students with the M-learning methodology. This correlation was weak for the “auscultation skills” (*p* = 0.032, R^2^ = 10.5%) and moderate for the “general knowledge and other competencies” (*p* = 0.001, R^2^ = 23.6%), Figure 5.

### 3.7. Feedback from Participants

**Figure 6:**
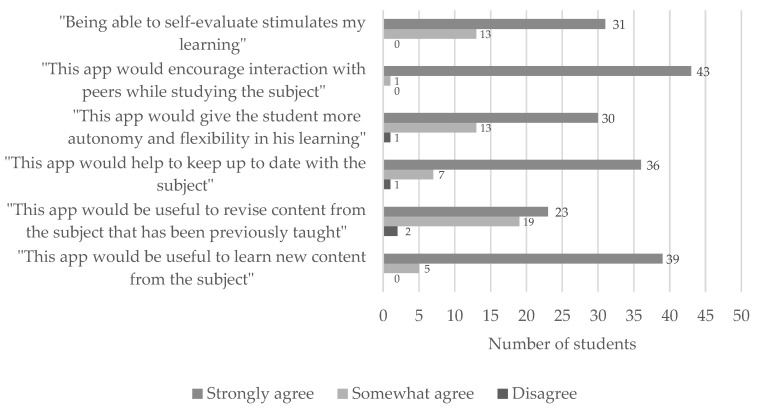
The qualitative approach to the usefulness of the app. Students answered a battery of questions expressing their degree of agreement with the different statements above.

In the final survey, the participants answered some further questions to better define the benefits and limitations of the new tool. In doing so, they referred to their degree of agreement with several statements, as shown in Figure 6.

## 4. Discussion

In this work, we have comprehensively evaluated the use of a mobile app to support remote learning by making it accessible to a sample of medical students transitioning to their clinical years. 

As the main objectives of the study were based on the self-perception of the students and not the academic results, we had to ensure that the participants understood the new tool to reach valuable subjective conclusions [35]. The rate of understanding of our project among students was higher than those obtained in other studies [41], reaching a 100% of students who understood the aims and scope of the study and the purpose and utilities of the app. 

In our cohort, there is a significantly higher proportion of women (72%) than men, but that correlates with the epidemiological data from medical schools in Spain, indicating that 70% of the medical students are women [42]. 

The main purpose of our intervention was to offer the students a new tool to supplement remote learning, ideally enhancing skills and knowledge acquisition outside of the clinical environment. This is something other authors have attempted by creating online classrooms [43] and syllabi as emergency alternatives to traditional hands-on education. However, as online resources only, their popularity is expected to fade as communities recover and full access to the clinical field returns [14]. On the contrary, our project has been envisioned long-term, as a supplementary rather than replacement tool [15,28], similarly to what some institutions have pursued regarding social networks and the new telemedicine programs [9], considering that they are here to stay and should be incorporated into curricula [29]. It is highly indicative of its utility, that around 85% of the students rated the potential learning benefits of the app at 8/10 or above, for both “auscultation skills” and “general knowledge and competencies of the subject”. However, we acknowledge that this approach is not enough to fully characterize the learning value of the app. Further research should ideally compare the outcomes of being trained with this app to those obtained with the traditional methodology, preferably through a prospective and randomized approach with two cohorts running in parallel [34]. What is more, further studies should dive deeper into the already suggested benefits of including team-based activities in these methodologies as we have [44], taking into account that students’ motivation is an important conditioning factor to learning [45].

The auscultation module displaying a virtual thorax, considered the core of the interactive part of the app, could either be used (1) to hear and learn the different heart sounds—physiological and pathological; or (2) to evaluate the student’s skill in recognizing a presented sound and identifying the underlying diagnose. Simulators successfully provided good correlation between the users’ results and their previous experience and knowledge [46]. Additionally, simulators’ benefits for the acquisition of knowledge and skills have been well characterized [34], ranging from auscultation skills among junior doctors [47] to highly technical ones in demanding surgical specialties [2]. This is why there has been a long tradition of their use in the history of Medical Education [48,49,50]. What is more, simulators have also proved their effectiveness for building confidence among trainees [51]. As a consequence, they have been proposed as an interesting substitute for patient interaction in these difficult times [12]. Nevertheless, the combination of good accessibility (especially of mobile apps) and a good capability of improving learning means that simulators in every format can become an interesting teaching supplement in the near future, regardless of the environment in which the teaching would have to be conducted [7,13].

The context in which the new tool is used and evaluated is critical for understanding its potential but analyzing its intrinsic characteristics might help us predict the extent of its utility under other circumstances [37]. We have evaluated the different characteristics of the app through a qualitative approach, as shown in Figure 6. Our initiative attracted encouraging feedback from students, who coincided in highlighting benefits already suggested by other authors for M-Learning schemes, such as the opportunity to self-evaluate through tests [26,52], the greater autonomy [21], the possibility to interact with other students [53], and higher flexibility in their learning process [54], facilitating the task of keeping up-to-date with the subject. A majority of our participants considered the app useful not only for revising and consolidating knowledge [55] but also for learning new topics [22]. Last but not least, the encouragement of a student-centered teaching methodology enhances the acquisition of other soft skills such as time management [56], although this may mainly benefit high achieving students, according to some authors [57].

On the other hand, it should be noted that when it comes to university staff, the app allows our personnel to follow the students beyond lectures halls and hospital wards, tracking their progress in the acquisition of capacities, and identifying potential problems in specific lectures where students show a poorer performance when answering the multiple-choice question of the app [24]. To wit, the interaction between students and teachers has been highlighted by other authors as one of the most significant parameters to take into account when designing M-Learning solutions [19,28].

Surprisingly, we have not found differences in the perceived utility of the app for improving skills and acquiring knowledge depending on the previous self-reported degree of digital competence, as other authors had previously reported [58]. This might be explained by the high proportion of students in our sample who reported high proficiency, which might as well be contextualized by the increasing demands of the upcoming generations for the new technologies as learning tools [15]. Another hypothesis supporting this fact is that the app was easy to follow, built with very important participation of students in its conception and development, through focus group interviews, and, thereby, adapted to their use [17,28].

One of the most recent reviews in the field of integrating technology into teaching methodologies could only include four studies comparing postintervention satisfaction between ‘M-Learning’ and traditional schemes. In that review, no statistically significant differences were found, and the evidence was not considered of high quality [33]. Despite being in an extraordinary situation, we have managed to contrast the opinions of the same set of students regarding the two models, and we have found that a significant improvement in satisfaction will be obtained if the app is kept as an additional resource. Indeed, previous research had already suggested that the most beneficial approach to the implementation of these new tools would be to consider them just supplementary to traditional lectures and clinical placements, the cornerstone of Medical Education, promoting a guided pathway for their use [52,59]. Another factor to note here is that the setting in which the app was used was during the SARS-CoV-2 pandemic, where teaching was not conducted face-to-face. Therefore, satisfaction, both with and without the app, may have been shifted to lower values than normal.

Admittedly, an increase in students’ satisfaction has also been described by including collaborative and teamwork activities in a subject’s curriculum [22,44]. This might be based on the activity theory approach and could be one of the key components that support the differences observed in our study. The app has allowed our group to register the participation of students in collaborative activities and identify their individual contributions, something that was complex to evaluate until now despite being considered compulsory in most of the subjects’ curricula since the implantation of the European Higher Education Area (EHEA). What is more, according to some studies, students expected online lectures and live broadcasts as the replacing teaching strategies in the COVID-19 era, rather than innovative digital tools [7], so this novel and unexpected approach might have helped to increase students’ satisfaction. 

Before this work, it has also been suggested that there is not enough evidence to consider cost-effective M-Learning methodologies [33]. However, one of the strengths of our work is that the app could be downloaded directly into the student’s smartphone, and therefore, the expenses of the project are limited to the development and maintenance of the new software. This approach could be more beneficial, given the speed at which the technological field is evolving, in preventing large investments in devices that will become outdated in a short time [9,20,27]. On the other hand, using students’ preowned devices for teaching purposes might also generate distractions [25,28], and ethical discussions should be held if sensitive information from patients is included [53]. Consequently, apart from new infrastructures and virtual platforms specific to this purpose, we want to emphasize the need for a strong policy of use [17,35]. Additionally, we should also bear in mind that, as with any new technological initiative, technical issues are expected. This can be more troublesome in some countries where the economic differences among students might be bigger, being those from poorer or more complex backgrounds more heavily affected by the challenges of implementing a system based on technology (e.g., the need for a high-speed internet connection) [15,60]. 

Limits of this study to be acknowledged are that the two teaching methodologies (traditional and M-Learning) that have been used for contrasting student’s satisfaction had not been put in practice in parallel nor for the same module of the subject, and therefore the opinions about the traditional methodology might be subject to recall bias. The response rates were just above 60% of the registered users, and although this has to be contextualized in the extraordinary lockdown situation that we were living at the time of collecting the data (leading to the disconnection of the students from the university environment), we have to consider the possibility of a nonresponse bias, meaning that those who completed the survey might have engaged more with the technology. Nevertheless, other studies carried out in the same pandemic context have shown much lower responses rates, so we are proud of the engagement generated [15]. In our case, all participants were volunteers, and new studies should avoid the potential inherent bias by making the new tool accessible to the total of students enrolled in the course. As stated before, this quasi-experimental pilot study should serve as the starting point, and we encourage peers to run new fully experimental studies with a randomized approach, cohorts running in parallel prospectively, and considering baseline characteristics, so as to avoid the influence of any possible confounding variable. Blinding was not possible either.

Despite considering the rigorous evidence here provided, its extrapolation to other settings and contexts has to be performed carefully as it comes from a pilot experiment and from a tool that has been optimized to the specificities of the local context [61]. The correlation between students’ expected satisfaction with an M-learning methodology and the learning value attributed to the app was weak but enough to significantly remark the complementarity of both parameters. As a consequence, we encourage peers to consider each of these parameters alongside others in future analyses. Further research could help to better define the influence of other secondary descriptive variables in the evaluation of the app by the students and to clarify the extent to which each of the components of the app (such as the collaborative activities) explain the high potential value attributed to it and the expected increase in the degree of satisfaction if the tool is finally adopted. Other uses of new technologies arising due to COVID-19, such as “Mobile Assessment” or “M-Assessment” (the natural evolution of M-Learning), are yet to be characterized [28]. 

## 5. Conclusions

In conclusion, this work has proved the feasibility of creating a mobile app to serve as an additional tool in medical teaching. In our work, students attributed a high learning potential to this app both for acquiring the “general knowledge and competencies” and for “clinical skills” of the subject being taught. Importantly, the creation of a mobile app as an adjunct to regular teaching resulted in a significant increase in the degree of satisfaction with the teaching methodology. Finally, the benefits of the new tool seem to be independent of the self-reported degree of previous digital competency reported by the students.

## Figures and Tables

**Table 1:** Volunteers’ demographics and the number of responses.

	Volunteers	
Male, *n* (%)	19	(27.5)
Female, *n* (%)	50	(72.5)
Total, (sex ratio F/M)	69	(2.6)
	Answered to the survey	
Male, *n* (%)	13	(29.5)
Female, *n* (%)	31	(70.5)
Subtotal “*n*”, (% from volunteers)	44	(63.7)

**Figure A1:**
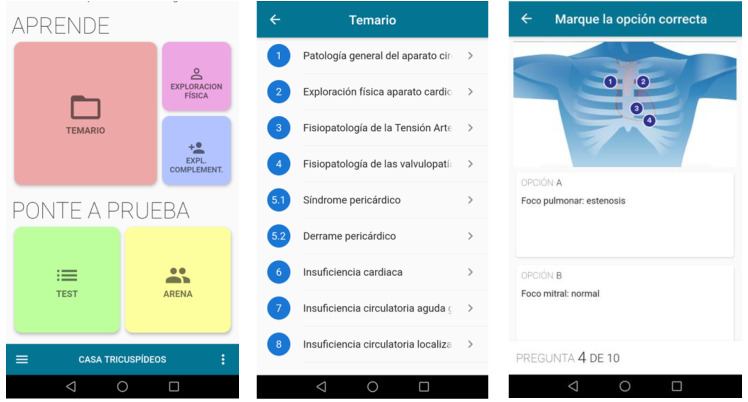
From left to right: “Sections”, “Lectures” and “Auscultation module”.

**Figure A2:**
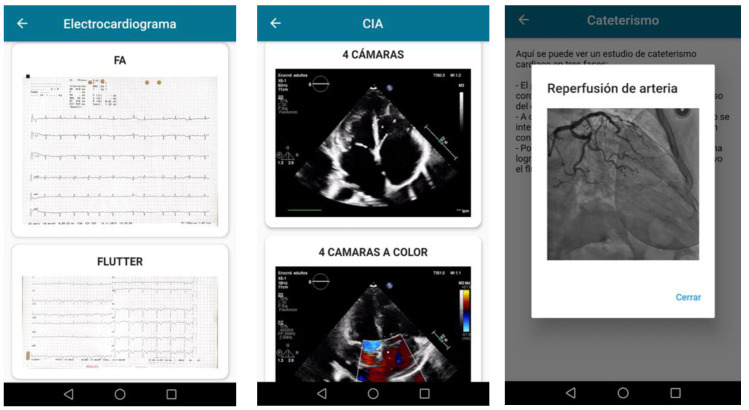
From left to right: “Electrocardiography”, “Echocardiography” and “Catheterization studies”.

**Figure A3:**
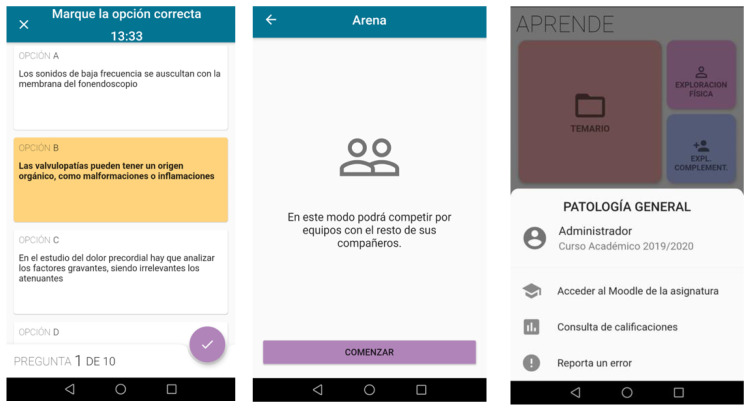
From left to right: “Test (MCQ)”, “Team-based competition” and “Other resources”.

## Data Availability

Anonymized data is available from the corresponding author, J.D.-L., P.P.-M., upon accepted reasonable request and under a collaboration agreement.

## Supplementary Materials

The following are available online at https://www.mdpi.com/article/10.3390/ijerph19052777/s1. An explanatory video of the app showcasing its different modules and functionalities can be found at: https://www.youtube.com/watch?v=uLWLIYK55E0 (Spanish audio, last accessed 19 February 2022).

## Appendix A. Description of the App

The materials and contents included in the app were either created or carefully selected from public repositories of interest. These included lectures presentations, tests, results from investigations, physiological and pathological recorded heart sounds, etc. The different modules of the app were:

1. Bookshelf. Presentations and resources connected to the lectures;
2. Physical exploration module. Useful information and didactic resources, presented in several formats (images, texts, videos, sounds, links) aiming to contribute to the teaching of the systematic process of exploring a patient. The app included an interactive module of auscultation in which the students could be trained in this set of skills following two approaches: the first one allowed them to select what kind of sound they wanted to hear, and the second one consisted of a multiple-choice battery of questions based on presented sounds on the virtual patient’s thorax. More than 25 physiological and pathological sounds were included;
3. Investigations. More than 30 resources and multi-format examples about electrocardiography, echocardiography, and catheterization studies were included;
4. Test. More than 1300 true and false statements filtered by the different topics covered in the subject were included to be randomly combined according to the students’ preferences when loading a self-assessment test;
5. Arena. Multi-participant and collaborative competition among the students. When registered, the students were assigned a team or “House”. In each of the 15 min programmed activities, the students answered multiple-choice questions to gain points for their “House”;
6. Others. Access to the University’s online platform, previous results, help, and support;

Images of the app:

## Appendix B. Survey Used

1. Rate your satisfaction with the methodology traditionally used for teaching the subject (e.g., the one used during the Hematology module).
  Totally unsatisfied                          Totally Satisfied
2. Indicate your expected satisfaction with a methodology combining both the traditional resources and the new app as it has been used during the Cardiology module.
  Totally unsatisfied                          Totally Satisfied
3. Up to what extent an app like this one could help you improve your auscultation skills and ability to identify heart sounds?
  Useless                                Really Useful
4. Up to what extent an app like this one could help you improve your knowledge and other competencies included in the “General Pathology” subject?
  Useless                                Really Useful
5. Rate your level of agreement with the following statement: “*My digital competence is good and I can use new technologies with ease*”.
  O Disagree.         O Somewhat agree.         O Strongly agree.
6. Have you received enough information about the app and the ongoing project, and do you fully understand its aim?
  O Yes.                                O No.
7. Rate your level of agreement with the following statements:
  	**Disagree**	**Somewhat Agree**	**Strongly Agree**
  “Being able to self-evaluate stimulates my learning”			
  “This app would encourage interaction with peers while studying the subject”			
  “This app would give the student more autonomy and flexibility in his learning”			
  “This app would help to keep up to date with the subject”			
  “This app would be useful to revise content from the subject that has been previously taught”			
  “This app would be useful to learn new content from the subject”			
