# Supplementary material for: A Pilot Study on the Feasibility of Developing and Implementing a Mobile App for the Acquisition of Clinical Knowledge and Competencies by Medical Students Transitioning from Preclinical to Clinical Years
Source: Int J Environ Res Public Health. 2022 Feb 27;19(5):2777. doi: 10.3390/ijerph19052777 (PMC8910514; doi:10.3390/ijerph19052777)
Supplement: Supplementary file 1 [file ijerph-19-02777-s001.zip › ijerph-1585399-supplementary.pdf]

Supplemental File

A Pilot Study on the Feasibility of Developing and Implementing a Mobile App for the Acquisition of Clinical Knowledge and Competencies by Medical Students Transitioning from Preclinical to Clinical years.

Appendix 1 – Description of the app

The materials and contents included in the app were either created or carefully selected from public repositories of interest. These included: lectures presentations, tests, results from investigations, physiological and pathological recorded heart sounds... The different modules of the app were:

1. Bookshelf. Presentations and resources connected to the lectures.

2. Physical exploration module. Useful information and didactic resources, presented in several formats (images, texts, videos, sounds, links...) aiming to contribute to the teaching of the systematic process of exploring a patient. The app included an interactive module of auscultation in which the students could be trained in this set of skills following two approaches: the first one allowed them to select what kind of sound they wanted to hear, and the second one consisted of a multiple-choice battery of questions based on presented sounds on the virtual patient's thorax. More than 25 physiological and pathological sounds were included.

3. Investigations. More than 30 resources and multi-format examples about electrocardiography, echocardiography and catheterization studies were included.

4. Test. More than 1300 true and false statements filtered by the different topics covered in the subject were included to be randomly combined according to the students' preferences when loading a self-assessment test.

5. Arena. Multi-participant and collaborative competition among the students. When registered, the students were assigned a team or "House". In each of the 15-minutes programmed activities, the students answered multiple-choice questions to gain points for their "House".

6. Others. Access to the University's online platform, previous results, help and support...

Supplemental File **: A Pilot Study on the Feasibility of Developing and Implementing a Mobile App for the Acquisition of Clinical Knowledge and Competencies by Medical Students Transitioning from Preclinical to Clinical years.**

Images of the app:

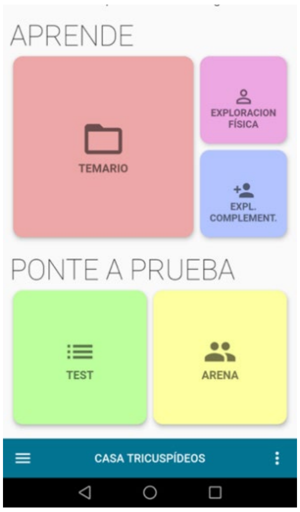

Sections

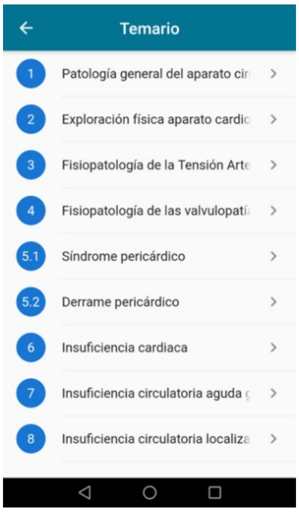

Lectures

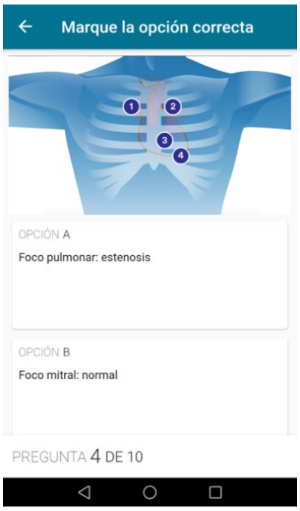

Auscultation module

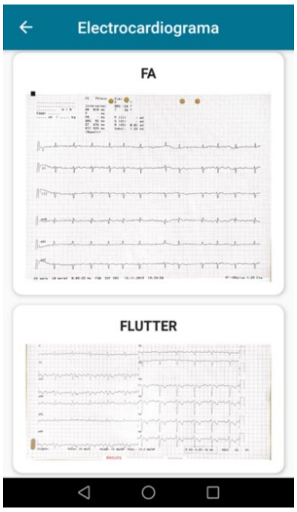

Electrocardiography

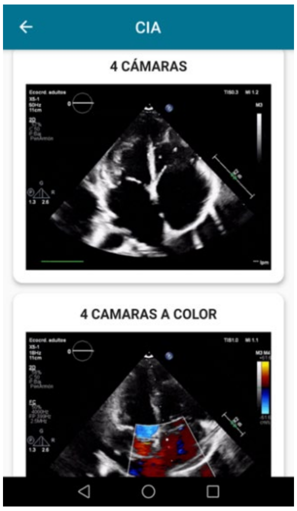

Echocardiography

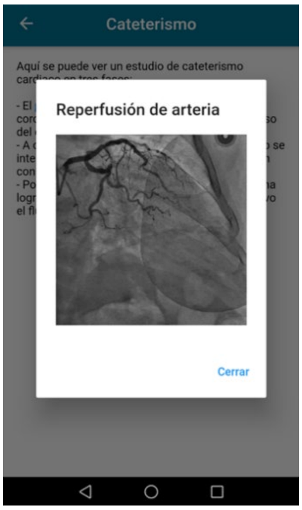

Catheterization studies

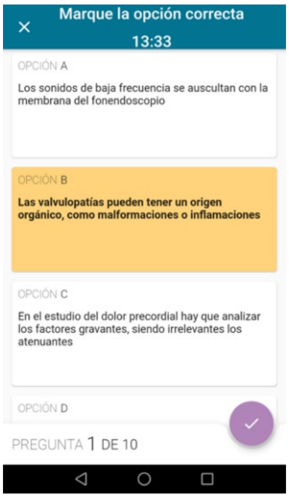

Test (MCQ)

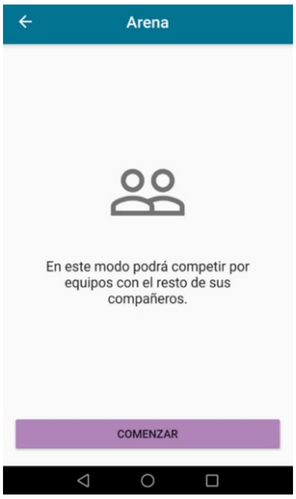

Team-based competition

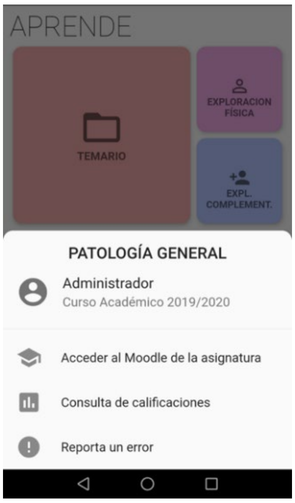

Other resources

Appendix 2 – Survey used

1. Rate your satisfaction with the methodology traditionally used for teaching the subject (e.g. the one used during the Hematology module).

Totally unsatisfied — Totally Satisfied

0	1	2	3	4	5	6	7	8	9	10

2. Indicate your expected satisfaction with a methodology combining both the traditional resources and the new app as it has been used during the Cardiology module.

Totally unsatisfied — Totally Satisfied

0	1	2	3	4	5	6	7	8	9	10

3. Up to what extent an app like this one could help you improve your auscultation skills and ability to identify heart sounds?

Useless — Really Useful

0	1	2	3	4	5	6	7	8	9	10

4. Up to what extent an app like this one could help you improve your knowledge and other competencies included in the "General Pathology" subject?

Useless — Really Useful

0	1	2	3	4	5	6	7	8	9	10

5. Rate your level of agreement with the following statement: ***"My digital competence is good and I can use new technologies with ease".***

O Disagree. O Somewhat agree. O Strongly agree.

6. Have you received enough information about the app and the ongoing project, and do you fully understand its aim?

O Yes. O No.

7. Rate your level of agreement with the following statements:

	Disagree	Somewhat agree	Strongly agree
"Being able to self-evaluate stimulates my learning"			
"This app would encourage interaction with peers while studying the subject"			
"This app would give the student more autonomy and flexibility in his learning"			
"This app would help to keep up to date with the subject"			
"This app would be useful to revise content from the subject that has been previously taught"			
"This app would be useful to learn new content from the subject"			
